# Saving the locals: a conservation genomics approach to the endangered Spanish Toothcarp, *Aphanius iberus* (Valenciennes, 1846)

**DOI:** 10.1038/s41598-025-31909-y

**Published:** 2025-12-11

**Authors:** Maria Estarellas, Alfonso López-Solano, Gabriel Mochales-Riaño, Silvia Perea, Adrián Talavera, Bernat Burriel-Carranza, Tessa Lynn Nester, Sergi Tulloch, Nati Franch, Josep Xarles, Jordi Ruiz-Olmo, Enric de Roa, Ignacio Doadrio, Salvador Carranza

**Affiliations:** 1 Institute of Evolutionary Biology (CSIC-Universitat Pompeu Fabra), Passeig Marítim de la Barceloneta, 37, 08003 Barcelona, Spain; 2 Museo Nacional Ciencias Naturales (CSIC), C. de José Gutiérrez Abascal, 2, 28006 Chamartín, Madrid, Spain; 3 New York University Abu Dhabi, Abu Dhabi, United Arab Emirates; 4 Museu de Ciències Naturals de Barcelona, Passeig Picasso s/n, Parc Ciutadella, 08003 Barcelona, Spain; 5 Parc Natural del Delta de l’Ebre (Generalitat de Catalunya), Av Catalunya, 46, 43580 Deltebre, Spain; 6 Fundació Zoo Barcelona, Passeig Picasso, 36, 08003 Barcelona, Spain; 7 Direcció General de Medi Natural I Biodiversitat (Generalitat de Catalunya), Carrer del Foc, 57, 08038 Barcelona, Spain; 8 Consorci per la protecció i Gestió dels Espais Naturals del Delta del Llobregat, Barcelona, Spain

**Keywords:** Genomics, Translocations, Population structure, Genetic diversity, *Aphanius iberus*, Ecology, Ecology, Evolution, Genetics

## Abstract

Understanding the genetic structure and evolutionary history of endangered species is crucial for effective conservation planning. The Spanish toothcarp, *Aphanius iberus* (Valenciennes, 1846), an endemic and euryhaline fish of the Mediterranean coast of the Iberian Peninsula, is currently threatened by habitat destruction, climate change, and anthropogenic translocations. Here, we employed both a single genetic marker (cytochrome b) and genome-wide SNP data from medium-coverage whole genomes to investigate the population structure, genetic diversity, and demographic history of *A. iberus*, especially focussing on its northern distribution, which has remained poorly studied. Our analyses revealed a well-structured genetic pattern across the species’ range, with four main genetic lineages: Northern Catalonia, Southern Catalonia, Levantine, and Murcian. Genomic indicators, including heterozygosity, ROHs, and migration analyses, suggest higher inbreeding and genetic erosion in the northernmost populations, likely due to long-term isolation, whereas southern populations maintain higher genetic diversity. We also identified several admixed and potentially translocated populations. These findings underscore the importance of accurately determining the origin of populations before any translocation or reintroduction, as misguided management may compromise the genetic integrity of native lineages. This work provides essential genomic insights to guide conservation strategies and emphasizes the need for lineage-aware management of endemic species like *A. iberus*.

**Supplementary Information:**

The online version contains supplementary material available at 10.1038/s41598-025-31909-y.

## Introduction

Conservation initiatives involving fauna and flora are frequently enhanced nowadays by a comprehensive integration of genomics^1,2^. Further insight in this area has the potential to optimize the allocation of financial resources and facilitate the implementation of more effective conservation strategies^3^. Concerning these decision-making processes, population genomics emerges as a powerful tool, as it focuses on the study of genomic variation within and among populations, as well as the evolutionary factors shaping this variation^4,5^. The results may be used to characterize the genomic profile of populations, elucidating their evolutionary history and, for example, enabling their classification into different Operational Conservation Units (similar to Designatable Units (or DUs)) for conservation management purposes^6–8^. Genomic data also provide information on connectivity, genetic diversity, inbreeding, and signatures of local adaptation. Combining approaches such as structure inference, phylogenomic reconstructions, migration modelling, and demographic history analyses offer a comprehensive view of population dynamics, evolutionary trajectories, and vulnerabilities, thereby supporting evidence-based conservation strategies^9–11^.

The application of these techniques is especially valuable for continental aquatic fauna, where dispersal and colonization are limited by geographical barriers^12^. While research has often focused on model species, domestic animals, or charismatic taxa^13^, many species with unique evolutionary traits remain overlooked, even when they can adapt to highly variable habitats. One such trait is euryhalinity, an ancestral adaptation that allows fish to thrive across contrasting salinity regimes but is retained by only ~ 3–5% of fish species^14–17^. This limited occurrence suggests that the physiological plasticity associated with euryhalinity entails significant costs, yet these species have attracted considerable scientific interest and serve as key models in ecological and physiological research^18–23^.

**Fig. 1:**
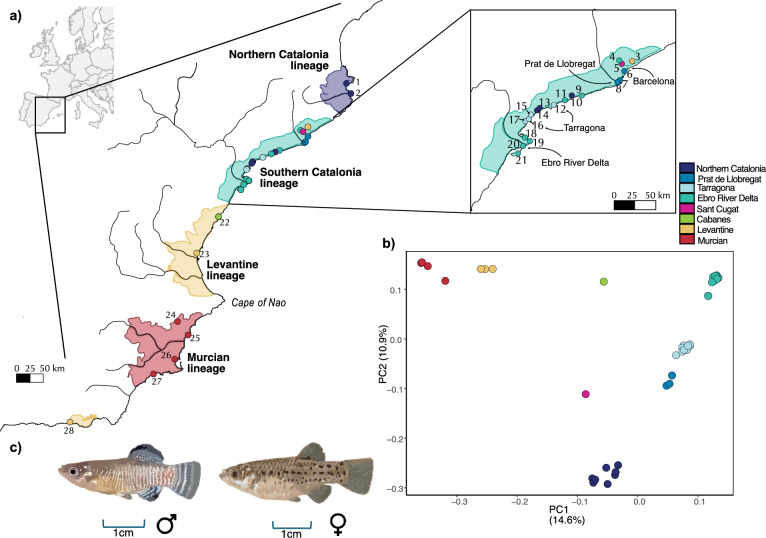
(**a**) Distribution of the populations of *A*. *iberus* included in the study. Genetic lineages are highlighted, and localities are colored according to the results of the PCA. (**b**) PCA analysis of *A*. *iberus*. (**c**) Representation of male and female individuals of *A*. *iberus*.

The Spanish toothcarp, *Aphanius iberus* (Valenciennes, 1846) (Fig. 1c), is an endemic species to the Mediterranean coast of the Iberian Peninsula, currently found in a wide range of habitats, including groundwater springs (locally known as *ullals*), coastal lagoons, river mouths, salt marshes, and even salt evaporation ponds where salinity can reach twice or more than that of seawater^6,24,25^. Notably, *A. iberus* and close relatives (e.g. *A. baeticus*) retain true euryhalinity, enabling movements across steep salinity gradients and potentially conferring resilience under ongoing climate-driven salinization of Mediterranean basin wetlands^15,26–30^. The species is currently Listed as Endangered in the Spanish National Catalogue of Threatened Species (RD 139/2011^31^), its populations face isolation and fragmentation due to habitat destruction, introductions of invasive species, translocations, and climate change, alongside other documented pressures on the species^32,33^. The species’ limited natural dispersal is mainly triggered by extreme weather events, such as intense storms and floods, often linked to Isolated High-level Atmospheric Depressions (locally known as *DANAs*), and, less frequently, by strong coastal waves^33,34^. These events, which are becoming more frequent under current climate change, can mobilize large volumes of water, transporting individuals across fragmented habitats and influencing the species’ biology and ecology. At the same time, global warming is not only leading to progressively warmer and saltier conditions, particularly in southern Spain, but also causing severe desiccation in many habitats^35,36^, making reintroduction and translocation strategies especially relevant in the species’ conservation management^33,34,37,38^.

The geographic distribution of the Spanish toothcarp has been previously studied, revealing a well-structured genetic pattern composed of several distinct lineages that reflect the spatial segregation of the species across its natural range (Fig. 1a)^6,11,25,33,39,40^. These lineages include: (1) the Northern Catalonia lineage, comprising two populations from Girona province, near the French border; (2) the Southern Catalonia lineage, which extends southwards to the Ebro River Delta; (3) the Levantine lineage (corresponding to Iberian Levante), represented by a single genetic population covering most of the Valencian region; and (4) the Murcian lineage (already referred to as such in Doadrio et al. 1996), stretching from Cape of Nao (southern Alicante province) through the province of Murcia to Rambla Moreras, the southernmost limit of the species’ range (Fig. 1a)^6,25,39^. Subsequent studies have refined this geographical and genetic gradient, identifying admixed populations located between these genetically distinct lineages, which show signs of historical or ongoing gene flow and may have important implications for the genetic diversity and evolutionary history of the species^7,11,33,40–42^. Translocations and reintroductions of *A. iberus* have occurred across its range, often without fully resolved source origin^11,25^. For example, Adra (locality 28) likely derives from Albuixech (loc. 23), the sole genetic representative of the Levantine lineage and a donor for multiple reintroductions^11^ (Fig. 1a); by contrast, Prat de Llobregat (locs. 7–8) was reintroduced in the mid-1990s with aquarist stock after a 1960s local extinction^43,44^, whereas Ebro Delta populations (locs. 18–21) are native and stable, aside from limited LIFE (1996–1999) experimental releases of captive-bred fish. Such actions can aid recovery but, without prior genetic/ecological vetting, risk eroding lineage integrity and local adaptation^32,45^ (Fig. 1a). These concerns are acute for populations persisting only in artificial habitats (e.g. Sax; loc. 24), where fitness and long-term resilience are especially vulnerable.

A primary objective of this study was to reconstruct and understand the evolutionary history and population structure of *Aphanius iberus* along its distribution range, with particular emphasis on the Catalonia region (Northern and Southern Catalonia lineages), located in the northward region of the species’ distribution range and which has not been extensively studied. We hypothesize that the present distribution of genetic lineages reflects a combination of historical isolation and recent anthropogenic influences, including translocations and habitat fragmentation. Given the well-documented phenomenon of mito-nuclear discordance, where mitochondrial and nuclear markers can reveal different phylogenetic relationships^3,46–48^, we applied next-generation sequencing (NGS) to generate genome-wide SNP data, enabling us to assess whether genomic-based population relationships align with those previously inferred from mitochondrial data, while providing a more comprehensive view of genomic diversity and lineage origins. Specifically, we used medium-coverage whole-genome sequencing (WGS), mapping reads to the reference genome and calling high-quality biallelic SNPs across individuals to obtain a dense, genome-wide nuclear panel suitable for both evolutionary inference and conservation-relevant comparisons. This study included the most representative conservation units from all lineages of *A. iberus*, as updated by Nester et al.^11^, together with nearly all currently documented populations from the species’ northern distribution range. This framework allowed us to identify populations of allochthonous origin and to confirm the natural status of others whose provenance has been debated. Further, we also contribute with valuable resources to for more targeted conservation plans such as analyzing the demographic history of the species and the level of variability, and adaptability of the genome. By applying a conservation genomics approach to this non-model, endangered species, we aim not only to inform region-specific management actions but also to contribute to the broader understanding of how genomic tools can enhance conservation strategies. This approach, centered on preserving the genetic identity and evolutionary legacy of local populations, underscores the value of “saving the locals” as a guiding principle for biodiversity conservation in fragmented and rapidly changing ecosystems.

## Results

### Sample selection and dataset construction

The combination of both the 174 newly generated and the 192 sequences for the Cytochrome b gene obtained from GenBank, resulted in an alignment of 985 base pairs. The haplotype network revealed 75 unique haplotypes for *A. iberus* and an additional five haplotypes for its closest relative, *A. baeticus* (Table S2, Fig. S1). Within *A. iberus*, haplotypes from the Murcian lineage formed two main clusters: one comprising coastal localities 25–27 and 30–41 (Mar Menor, Santa Pola, Vinalopó, Rambla Moreras, and Chícamo), and another corresponding to inland water localities 24 and 29 (Sax and Villena, respectively), in agreement with previous genetic studies^11,33^. The Levantine lineage, including the Albuixech population (locality 23) and the allochthonous population from Adra (locality 28), also formed a distinct cluster. In contrast, haplotypes from the northern Mediterranean coast (Northern and Southern Catalonia lineages) grouped into three clusters, encompassing 21 unique haplotypes. The Southern Catalonia lineage (localities 9, 11, 15–21) clustered closely with localities 6–8 from the Llobregat River Delta. While a second and third cluster grouped localities 12 and 17 (Tributaris–Sèquia Major and Torrent del Pi) and another one the Northern Catalonia lineage (localities 1, 2, 10, 13 and 14) (Table S2, Fig. S1).

A total of 46 *A. iberus* individuals were selected for WGS, ensuring representation of all mitochondrial haplotypes from localities 1–21 (Catalonia region), complemented by seven individuals from the remaining distribution (localities 22–28) and two outgroups (*A. baeticus* and *A. anatoliae*). DNA concentrations averaged 10 ng/µL (range: 6.5–20 ng/µL). Sequencing generated between 17,293,672 and 90,793,054 raw reads per individual (mean: 65,273,642), corresponding to 2.594–13.619 GB of data (mean: 9.791 GB) per sample. Reads were mapped to the already available reference genome^59^ and after duplicate removal, quality filtering, and exclusion of repetitive regions, Dataset 1 (all 46 individuals) comprised 7,248,998 SNPs, including 1,160,109 unlinked SNPs (uSNPs). Dataset 2 (restricted to the 44 *A. iberus* individuals) contained 8,952,228 SNPs, of which 1,339,194 were uSNPs.

### Population structure, phylogenomic reconstructions, and migratory events

#### Principal component analysis

The PCA revealed clear geographic structuring in *A. iberus* populations (Fig. 1b). PC1 (14.6% of the variance) separated the Levantine lineage (locality 23) and the Murcian lineage (localities 24–27) from the Catalonia region (Northern and Southern Catalonia lineages, localities 1–21). PC2 (10.9% of the variance) reflected a north–south gradient among Catalan populations. Therefore, based on the PCA, six genetic clusters were identified: three corresponding to previously described lineages (Levantine, Murcian, and Northern Catalonia) and three subclusters within the Southern Catalonia lineage: Ebro River Delta (localities 9, 11, 18–21), Tarragona (localities 12, 15–17), and Prat de Llobregat (localities 6–8). Several geographically discordant populations were also detected, such as Adra (locality 28) and Mollet del Vallès (locality 3) clustering with Levantine. Some populations, such as Cabanes (locality 22) and Sant Cugat del Vallès (locality 5), did not group with any cluster and occupied intermediate positions between clusters. Notably, several populations from the Southern Catalonia lineage (localities 10, 13, and 14) clustered genetically with the Northern Catalonia lineage.

#### Population structure with Structf4

**Fig. 2:**
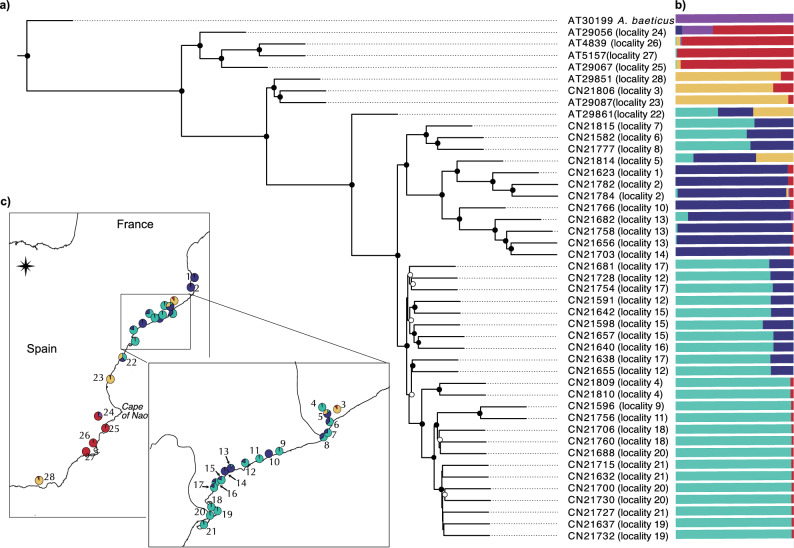
(**a**) Phylogenetic Tree inferred using a ML approach based on genomic data. Bootstraps > 95 are marked with a black circle and > 75 with a white one. The tree was rooted with *A.* *anatoliae*, but the branch is not shown for visualization purposes. (**b**) and (**c**) Structf4 K = 5 analysis as bar charts or on the map as pie charts.

The optimal *K* from the Structf4 analysis was determined to be 5 (Fig. S2), corresponding to the four main lineages (Northern Catalonia, Southern Catalonia, Levantine and Murcian) plus the outgroups (Fig. 2b,c). The Murcian lineage (localities 24–27) showed nearly homogeneous ancestry, except locality 24, which shared some ancestry with the outgroup. The Levantine lineage (locality 23) displayed an ancestry profile almost identical to Adra (locality 28) and Mollet del Vallès (locality 3). In Catalonia, two main ancestry components matched the two described lineages, with additional admixed groups. The Northern Catalonia lineage shares ancestry with southern localities 10, 13, 14. Southern Catalonia lineage shares ancestry with Rubí (locality 4), which is spatially distant. Two further admixed groups within the Southern Catalonia region corresponded to the Tarragona cluster (localities 12, 15–17) and the Prat de Llobregat cluster (localities 6–8), the latter showing slightly more Northern Catalonia ancestry along a geographic gradient. Tarragona itself was split into a northern group (locality 12) and a southern group (localities 15–17). Sant Cugat del Vallès (locality 5) exhibited a complex profile combining Levantine, Northern, and Southern Catalonia ancestry. Cabanes (locality 22) showed admixture between both Catalonia lineages and Levantine lineage, consistent with its geographic location (Fig. 1a).

#### Maximum likelihood tree with IQTree

The ML phylogeny, rooted with *A. anatoliae* (branch omitted in Fig. 2a), recovered *A. baeticus* as sister to all *A. iberus* samples, which formed five well-supported and geographically structured clades. The Murcian lineage was the first to diverge, followed by the Levantine lineage, both separated by Cape of Nao (Fig. 2c). Adra (locality 28) clustered with Levantine lineage rather than Murcian lineage, matching previous results^11^. Albuixech (locality 22) also forms a separate, highly supported clade, sister to the Northern and Southern Catalonia lineages, nevertheless, when looking at the output from Structf4 it doesn’t form an independent group but rather the result of the admixture of the different lineages. The Northern and Southern Catalonia lineages were reciprocally monophyletic, each forming subgroups consistent with the PCA and Structf4 results. The Northern Catalonia clade included the northernmost populations plus localities 10, 13, 14 and Sant Cugat del Vallès (locality 5). Some Southern Catalonia populations (e.g. Bassa Nova in Gaià, Madrigueres, and Rubí (localities 9, 11 and 4 respectively)) showed shared ancestry with more distant populations. Relationships among Tarragona and nearby populations (localities 12, 15–17) were less resolved, with shorter branches and lower support, suggesting lower divergence. The Prat de Llobregat population (localities 6–8) was phylogenetically closer to Northern Catalonia, consistent with introgression prior to its extirpation during the construction of the Barcelona Airport and later reintroduction.

#### Migration patterns

**Fig. 3:**
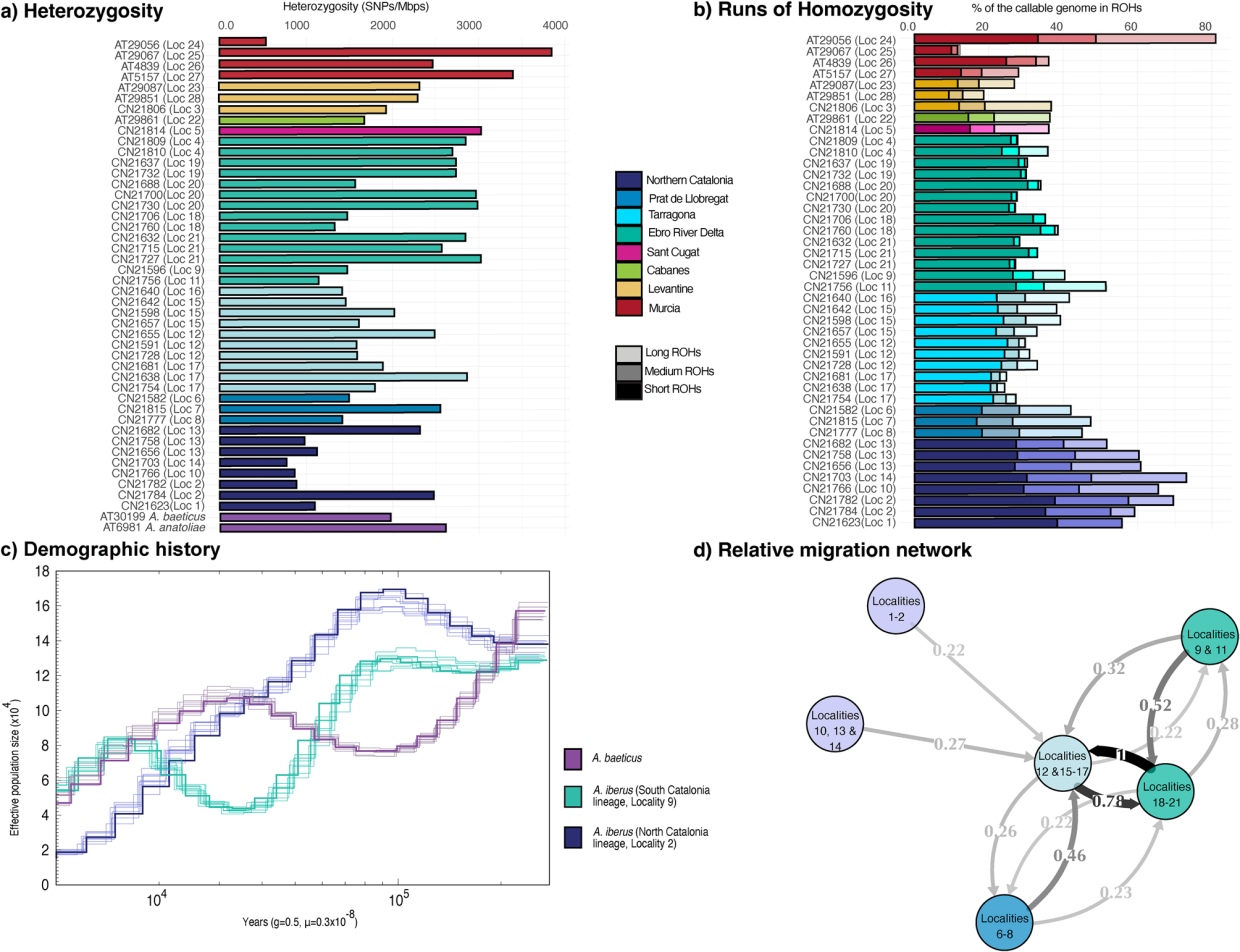
(**a**) Genome-wide heterozygosity levels of all individuals of *A*. *iberus* included in the study. (**b**) Percentage of the genome in Runs of Homozygosity (ROHs). (**c**) Demographic history of two populations of *A*. *iberus* and one *A*. *baeticus* during the Pleistocene. (**d**) Migration network (Effective Migrants per generation Nm) amongst the genetic groups identified in the population structure analyses.

Migration analysis with divMigrate revealed directional gene flow from the Northern Catalonia lineage (localities 1, 2, 10, 13, 14) towards Tarragona (localities 12, 15, 17) within the Southern Catalonia region, with no evidence of reverse flow. This indicates that the northern lineage acts as a genetic source rather than a recipient. In contrast, Ebro River Delta (localities 9, 11, 18–21), Tarragona (localities 12, 15–17), and Prat de Llobregat (localities 6–8) appeared genetically interconnected, suggesting recent or ongoing exchange among geographically close populations (Fig. 3d). The strongest exchange of migrants (> 0.75) is seen between Tarragona (localities 12, 15–17) and the localities situated at the Ebro River Delta (localities 18–21). Gst and Nm were identical, different selection of localities as well as Jost’s D migrations networks can be found in Fig. S3.

### Genome-wide diversity

A genome-wide assessment of heterozygosity was conducted for all individuals (Fig. 3a). The values varied substantially, both between and within populations, with most falling within the range of 1000 to 3000 heterozygous SNPs per megabase pair (SNPs/Mbp). The lowest heterozygosity was recorded in the Murcian lineage population of Sax (locality 24), with approximately 500 SNPs/Mbp. In contrast, the highest value was observed in the nearby population of Santa Pola (locality 25), with approximately 3,800 SNPs/Mbp. In Catalan lineages, the lowest heterozygosity levels were observed in the northern one. Conversely, the admixed Sant Cugat population (locality 5) showed the highest values in the region (3000 SNPs/Mbp). Populations from Southern Catalonia exhibited very diverse levels of heterozygosity (ranging between 1150 and 3000 SNPs/Mbp).

The analysis of ROHs showed consistent patterns across populations (Fig. 3b). As expected from its low heterozygosity, the Sax population (locality 24) exhibited approximately 80% of its callable genome within ROHs, with nearly 35% in long ROHs. In contrast, Santa Pola (locality 25) showed the lowest ROH percentage and virtually no long ROHs (both from the Murcian lineage). Among Catalan populations, those from the Southern Catalonia lineage: Ebro River Delta (localities 9, 11 and 18–21) and Tarragona (localities 12 and 15–17) had lower ROH proportions, primarily composed of short segments. In contrast, populations from the Northern Catalonia lineage displayed a higher percentage of the genome within ROHs, including a greater presence of long segments. The admixed populations: Tarragona (localities 12 and 15–17) and Prat de Llobregat (localities 6–8) did not exhibit particularly low ROH values. Localities 9 and 11 exhibited both a higher percentage and greater length of ROHs compared to other Ebro River Delta localities (18–21), likely reflecting their captive-breeding origin, despite their close geographic and genetic proximity. Individuals from localities 10, 13 and 14 had ROH profiles similar to those from the Northern Catalonia lineage (localities 1 and 2), from which they likely originated. Prat de Llobregat populations (Localities 6–8) in Barcelona exhibited around 40% of their genome in ROHs, but these occurred in longer stretches compared to most other populations.

### Demographic history

To infer the demographic history, we used a high-coverage dataset comprising 1,198,608,839 base pairs obtained from three individuals: two *Aphanius iberus* individuals, one from the Southern Catalonia lineage (locality 9) and one from the Northern Catalonia lineage (locality 2), and one individual of *Aphanius baeticus* as an outgroup. All three populations showed signs of demographic decline during the Pleistocene (Fig. 3c). In the Northern Catalonia lineage, this decline appears to have been continuous and uninterrupted. In contrast, the Southern Catalonia lineage experienced a partial demographic recovery after the initial decline, although recent trends indicate that the populations may be declining once again. A similar pattern was observed in *A. baeticus*, which also exhibited a phase of recovery followed by a renewed decline, paralleling the demographic trajectory of the Southern Catalonia lineage of *A. iberus*.

## Discussion

*Aphanius iberus* exhibits a distinctive geographic genetic distribution pattern, characterized by distinct genetic lineages and the intermingling of admixed populations^6,11,33^. This genetic structure reflects the extensive evolutionary history shaped by local adaptations to diverse environmental pressures yet remains vulnerable to anthropogenic disturbances such as non-native translocations. The reconstruction of the history of its populations was one of the primary objectives of this study. Taken together, analyses of population structure, phylogenetic relationships, genome-wide heterozygosity, ROHs, migration, and demographic history reveal a robust population structure indicative of long-established populations^6,44^. Additionally, the unique adaptability of *A. iberus* to varying salinity levels is believed to have evolved in response to historical climatic events, such as the Messinian salinity crisis^41^. During this period, drastic changes in the Mediterranean basin led to the formation of highly saline environments. The ability of the species to thrive in these fluctuating conditions reflects a costly yet effective evolutionary strategy, enabling them to become euryhaline and eurythermal^15,26–30^. This adaptability contrasts with the more stable habitat preference of other Mediterranean closely related cyprinodontiform (e.g.: *Valencia hispanica*) which occupy consistent freshwater environments and follow a less physiologically demanding, but potentially less versatile strategy^41,49,50^. However, despite its broad salinity tolerance, *A. iberus* exhibits very limited natural dispersal capacity across open sea, a constraint that has likely reinforced the historical isolation among lineages. These historical adaptations underscore the resilience of the species and highlight the evolutionary pressures that have shaped its current distribution and genetic diversity.

Our mitochondrial haplotype network, phylogenomic analyses and population-structure results resolved four main genetic lineages in *A. iberus*: Northern Catalonia, Southern Catalonia, Levantine and Murcian (Fig. 1a). These lineages refine the three major groups originally described by Doadrio et al.^6^ (Catalonia, Levantine and Murcian), with the former Levantine clade further subdivided into a Southern Catalonia lineage (Ebro River Delta populations, localities 18–21) and a Levantine lineage sensu stricto represented only by the Albuixech population (locality 23)^11,33^. Importantly, our genome-wide data also revealed genetically intermediate and admixed populations between the two Catalan lineages (localities 7, 8 and 12), which had remained undetected in previous works^11,33^. Contrary to the well-documented potential for mito–nuclear discordance^3,46–48^, our cytochrome b haplotype network and SNP-based analyses were highly concordant, reinforcing the robustness of these inferences. Using the mentioned genomic approaches, we could distinguished populations of native origin from those likely resulting from translocations. For example, some populations within the Southern Catalonia lineage (localities 10, 13, and 14) show introgression from the Northern Catalonia lineage, whereas the Prat de Llobregat (localities 7 and 8) and Salou (Tributaris–Sèquia Major, locality 12) populations likely have a native origin (Figs. 1, S1). In contrast, other populations deviated from the expected geographic genetic gradient, clustering instead with distant populations and sharing their ancestry, migration patterns, and heterozygosity levels; this was the case for most localities 9–17, excluding aforementioned locality 12. These results, integrated with historical and institutional records, provide a detailed framework for clarifying the origin of each population and guiding lineage-specific conservation and management actions (expanded in detail in the Supplementary Results & Discussion).

Our genome-wide analyses across the full range of the species, including populations within and outside Catalonia, refined previously described patterns of the geographic gradient of *A. iberus*. Phylogenomic relationships, PCA clustering and the haplotype network jointly recovered the expected large-scale structure but also clarified the position of several key populations (Figs. 1b, 2, S1)^6,7,11,33,41^. The StructF4 results identified the Cabanes population (locality 22) as admixed between the Levantine, Northern Catalonia and Southern Catalonia lineages. While previous studies classified this population as admixed between the Levantine and Southern Catalonia lineages, the current result may reflect the limited sampling in the dataset (only one individual was included, and intermediate populations were absent). Still, the phylogenetic tree places the Cabanes population (locality 22) as a sister branch to the Catalan lineages (Northern and Southern Catalonia) and the Levantine lineage, supporting previous findings. The same pattern was observed for the Murcian lineage: populations previously treated as separate conservation units collapsed into a single cohesive genomic cluster^11,33^. The Adra population (locality 28), historically considered the southernmost occurrence of the species, shows a clear genomic affinity to Albuixech (Levantine lineage, locality 23) rather than to neighbouring Murcian populations. Our results therefore independently corroborate the scenario proposed by Nester et al.^11^, in which Adra represents a translocation from Albuixech, a population that has undergone extensive captive breeding and now acts as a major donor for reintroductions. Similarly, the Mollet del Vallès population (locality 3) in the suburbs of Barcelona city appears to have originated from the same source (Levantine lineage).

Migration analyses, restricted to native populations to avoid translocation bias, revealed strongly directional gene flow from the Northern Catalonia lineage towards the south, with no evidence of reciprocal migration (Fig. 3d). These patterns likely reflect historical (multi-generational) gene flow, rather than current, ongoing migration. The absence of recent northward inflow suggests long-term isolation of the Northern Catalonia lineage, potentially reinforced by regional oceanographic and geographic conditions^51,52^. Along the Levantine coast, sediment accumulation from the Ebro River has generated a relatively wide and shallow shelf that could facilitate dispersal, whereas the much steeper and deeper shelf of Northern Catalonia may act as a natural barrier^53^. Furthermore, prevailing surface circulation in the western Mediterranean is dominated in this sector by a predominantly southward along-slope current, which may have further promoted past dispersal in that direction, despite localized seasonal reversals^51,52^. The current absence of populations between the Barcelona area (Prat de Llobregat, localities 7–8) and the Girona populations of Northern Catalonia (localities 1–2)^7,11,24,31,33^, creating a gap of roughly 70 km, may reflect a lack of suitable habitat or undocumented historical reductions in distribution. In contrast, high levels of interpopulation exchange were observed between the Ebro River Delta (localities 18–21) and Tarragona (localities 12, 15, 16 and 17), as well as with the Prat de Llobregat (localities 7–8), suggesting that historical connectivity existed among central-to-southern Catalan populations despite geographic separation.

The overall levels of genomic diversity in the Spanish toothcarp are relatively high for an Endangered species (ca. 1000–3000 SNPs/Mbp), especially when compared with other threatened vertebrates such as the Pyrenean desman (*Galemys pyrenaicus*), Tasmanian devil (*Sarcophilus harrisii*), and Sumatran orangutan (*Pongo abelii*), which show heterozygosity values as low as 12, 320, and 1200 SNPs/Mbp, respectively^3^. This is consistent with previous work highlighting the high genetic variability of *A. iberus*^6,11,25,33^. In our study, populations from the Northern Catalonia lineage (localities 1–2) displayed the lowest levels of heterozygosity, consistent with a likely southern origin of the species, as peripheral populations at the northern distribution limit often harbor reduced genetic diversity, and the highest ROH proportions, suggesting a decline in genetic diversity due to repeated bottlenecks and increased susceptibility to genetic drift. Conversely, introduced populations in the Barcelona province (such as localities 3, 4, and 5) showed high heterozygosity and low ROH levels. While these metrics may suggest high genetic diversity, they are likely artifacts of recent admixture, particularly in Rubí and Sant Cugat (localities 4 and 5), which show genetic signatures from the Southern Catalonia lineage. Other populations showing higher percentages of ROHs are localities 7 and 8, which were reintroduced from an ex-situ breeding program (currently locality 6). Besides presenting a larger amount of ROHs, it is notable that around 15% of the genome is in long stretches of homozygosity, an indicator of recent inbreeding loops likely caused by the captive breeding program^50^.

The demographic history analysis revealed a persistent decline Nₑ size through the Pleistocene, particularly in the Northern Catalonia lineage. In contrast, the Southern Catalonia lineage also experienced demographic contractions, but these were not continuous, with a period of recovery before a more recent decline. Although PSMC cannot directly infer dispersal, the sustained low Nₑ in the north is consistent with long-term isolation, as discussed in the migration section, where no evidence of recent incoming gene flow was detected (Fig. 3c,d). This isolation likely reduced opportunities for replenishment of genetic diversity, increasing the susceptibility of Northern Catalonia populations to genetic drift and inbreeding, as reflected in their higher frequencies and longer runs of homozygosity. Despite these demographic constraints, the species exhibits traits that may enhance its resilience, such as high reproductive output, rapid sexual maturation, and fast growth rates^7,44^. Supporting this, ROH patterns in southernmost populations (excluding locality 24) and, to a lesser extent, in Southern Catalonia indicate relatively good genomic diversity. ROHs in these groups are predominantly short, covering 20–25% of the genome, which is consistent with ancient, rather than recent, bottlenecks. In contrast, long ROHs are mainly observed in Northern Catalonia, consistent with smaller population sizes and mating between closely related individuals.

The role of anthropogenic translocations (whether intentional or accidental) is of particular concern, as they can disrupt natural evolutionary trajectories and erode locally adapted population structures. This is especially problematic for *Aphanius iberus*, a species with well-defined genetic lineages and likely local adaptations critical for survival. The translocation of non-native individuals may dilute these adaptations, thereby reducing fitness and resilience. Habitat transformation driven by agricultural expansion and coastal development further threatens population integrity. In highly anthropized environments, such as the irrigation ponds inhabited by the Sax population (locality 24), selective pressures differ markedly from those in natural habitats, potentially driving divergent evolutionary trajectories. Peripheral populations, such as those in Northern Catalonia (localities 1–2), face additional challenges due to their position at the range margin, where vulnerability is heightened. Conversely, our results suggest that some populations from the Levantine and Murcian regions may be better equipped to cope with rising temperatures and climatic variability. Nonetheless, a comprehensive conservation strategy must consider all populations, especially those in the north, to safeguard the species’ long-term resilience and genetic diversity. In light of our findings, the integration of high-resolution genomic tools emerges as essential to detect hidden admixture, resolve the origins of uncertain populations, and design targeted, locally adapted management strategies. Such approaches provide a robust framework for addressing historical uncertainties and ensuring the preservation of *A. iberus* in the face of ongoing environmental change.

## Materials and methods

### Sample collection, DNA extraction, and mitochondrial data screening

Tissue samples were obtained by collecting caudal fin clips, following standard protocols and in coordination with the relevant authorities. Adult individuals of *A. iberus* (average standard length 2–4 cm; body weight 0.5–1 g) were euthanized by immersion in an overdose of buffered tricaine methanesulfonate (MS-222, 0.1%), in accordance with internationally approved protocols for small cyprinodontiform fishes. All procedures were performed by qualified personnel under the responsibility of the competent authorities (Consorci del Delta del Llobregat, Parc Natural del Delta de l’Ebre, and Departament de Recerca i Universitats de la Generalitat de Catalunya), with collection and handling authorized within the framework of the research project SGR-00420.

The study protocol and methods were approved by the Animal Experimentation Ethics Committee of the National Museum of Natural Sciences (CEEA-MNCN; Spanish Animal Experimentation Research Centre no. ES280790000189), in strict accordance with current Spanish law (RD53/2013), transposed from European Union Directive 2010/63/EU (art. 2, 5f). All experimental protocols were conducted in accordance with relevant guidelines and regulations and are reported in compliance with the ARRIVE guidelines (https://arriveguidelines.org). Humane endpoints consisted of monitoring for the immediate loss of opercular movement and reflexes after immersion in the anesthetic solution, thereby confirming the effectiveness of euthanasia and safeguarding animal welfare.

Fin clips were stored in 95% ethanol and deposited in a − 80 °C freezer until processing. DNA was extracted using the DNeasy Blood and Tissue Kit (Qiagen) following the manufacturer’s specifications. DNA quantification was carried out through fluorometry using Qubit Broad Range (Thermofisher). Suitable DNA samples were then selected for sequencing. Detailed information about samples and locations can be found in Table S1.

In order to account for the full genetic diversity present in the Catalonia region (Northern and Southern Catalonia lineages), located along the northeastern coast of the Iberian Peninsula, we initially sequenced the cytochrome b gene for a total of 174 individuals using the primers GluF (5’ AACCACCGTTGTATTCAACTACA 3’) and ThR (5’ ACCTCCGATCTTCGGATTACAAGACCG 3’)^54^. Each reaction consisted of 12.5 µl of Speedy Supreme NZYTaq 2 × Green Master Mix (NZYtech), 9 µl of double-distilled H_2_O 0.5 µl of each primer and 1 µl of target DNA. PCR conditions consisted of an initial denaturation step of 5 min at 95ºC, followed by 41 cycles, each consisting of 2 s denaturation at 94ºC, 5 s of annealing at 55ºC, 5 s of extension at 72ºC and a final extension for 2 min at 72ºC. Newly generated sequences were supplemented with an additional 192 sequences obtained from NCBI GenBank (Sample information and GenBank accession numbers can be found in Table S2). The 366 sequences were aligned using MAFFT implemented in Geneious Prime v.2023.0.4. The resulting alignments were used to construct a haplotype network using the TCS Network Builder^55^ and the graph file was visualized using TCSbu^56^.

The haplotype evidence was used to maximize the genetic diversity of the sampling, a total of 44 samples of *A. iberus* were chosen for WGS. This included 37 individuals from the northern distribution of the species (Localities 1–21) and seven additional samples of *A. iberus* from across the species’ entire distribution range, ensuring representation of all four known genetic lineages previously identified, namely, Levantine, and Murcian lineages. Additionally, a sample of the sister species *Aphanius baeticus* (Doadrio, Carmona & Fernández-Delgado, 2002) was included as a closest related outgroup, while a sample of *Anatolichthys anatoliae* (Leidenfrost, 1912) was incorporated as a more distant outgroup (Fig. 1a).

### Data generation and processing

High quality DNA samples were then sent for Illumina library preparation and sequencing on a NovaSeq X Plus (PE150) targeting at least 10X coverage for 42 individuals and 15X for four samples (Table S1).

Raw reads were filtered, and adapters were removed using fastp^57^ with a minimum base quality score of 30, activating automatic adapter detection for paired-end sequencing and trimming polyG/X tails. To assure the quality of the data and correct trimming, sequences were checked with FastQC v.0.20.0^58^. Filtered reads were mapped against the reference genome (1.15 Gb) of *Aphanius iberus*^59^ with bwa-mem v0.7.17^60^ and sorted using Samtools v1.9^61^. Duplicate reads were removed using Picard^62^ and reads with a mapping quality below 30 were discarded using Samtools view. SNPcalling was done using Haplotypecaller from GATK^63^ with BP_resolution. Output files were combined using CombineGVCFs, and genotyping was carried out with GenotypeGVCFs from GATK^63^.

At this stage, we generated two different datasets: Dataset 1, containing all 46 individuals, including the two outgroups, *Aphanius baeticus* and *Anatolichthys anatoliae*; and Dataset 2, containing exclusively the 44 individuals of *Aphanius iberus.* This second dataset was used for analyses at an intraspecific level*.* Datasets are detailed in Table S1. Coverage was calculated and is detailed in Table S1. The four samples sequenced at higher coverage were downsampled to a similar coverage to the other samples using Picard^59^ for inclusion in Datasets 1 and 2.

The reference genome was scanned for repetitive regions, which were subsequently removed. Datasets 1 and 2 were filtered to exclude variants matching at least one of the following criteria: Quality by Depth (QD) < 10.0, Mapping Quality (MQ) < 55.0, Fisher Strand test (FS) > 50.0, StrandOddsRatio (SOR) > 5.0, MQRankSum < –5.0 && MQRankSum > 5.0, and ReadPosRankSum < –5.0 && ReadPosRankSum > 5.0. Further filtering of genotypes was performed using VCFtools, selecting variants with a quality score above 30, retaining only biallelic SNPs, removing indels, a minor allele frequency (MAF) of 0.001, and no missing data. To account for linkage disequilibrium, a secondary dataset was generated for each of the Datasets 1 and 2 retaining only unlinked SNPs (uSNPS) using bcftools with r2 ≤ to 0.5.

Collectively, the genomic datasets cover the species’ entire distribution, and the large SNP datasets enable comprehensive assessments of population structure, phylogenetic relationships, demographic processes, migration dynamics, and genomic diversity.

### Population structure and phylogenomic analyses

To gain a preliminary insight into the variability among the samples, a Principal Component Analysis (PCA) was conducted using Plink v1.9^64^ on *A. iberus* individuals (Dataset 2, unlinked SNPs). Additionally, we conducted a population structure analysis using Structf4^65^, considering 2–7 ancestral components and 50,000,000 iterations for both the first and second MCMC chains. Results were visualized with R v.4.3.2^66^. These analyses were performed on Dataset 1 and 2 (unlinked SNPs), which included the two outgroups to determine if some lineages were external.

To investigate phylogenomic relationships among populations, we constructed a maximum-likelihood (ML) phylogeny. To do so, we filtered Dataset 1 to exclude missing data and randomly selected one allele per genomic site (i.e. each SNP position in the alignment), resulting in a total of 5,808,219 SNPs. The dataset was then divided into non-overlapping 100 kbp windows, and each window was analyzed independently using IQ-TREE2^67^ with the GTR + ASC model and 1000 bootstrap replicates. The best-scoring trees from each window were subsequently combined using ASTRAL v5.7.8^68^.

### Genome-wide diversity

We estimated genome-wide heterozygosity for each individual in Dataset 1 (including both genera *Aphanius* and *Anatolichthys*; unfiltered). To do so, we divided the reference genome into non-overlapping 100 kb windows. Within each window, heterozygous sites were counted using vcfhetcount from vcflib^69^. A site was considered callable if it was not an indel and had a mapping quality score above 30. Results were plot in R v.4.3.2^66^.

Runs of Homozygosity (ROHs) were calculated for each individual of *A. iberus* based on the density of heterozygous sites in the genome using the implemented Hidden Markov Model (HMM) in the bcftools roh function with the default parameter –AF-dflt 0.4^70^, on the filtered Dataset 2. *A. baeticus* and *A. anatoliae* were excluded from this analysis to prevent them from influencing the results by causing noise, as the patterns of homozygosity can vary considerably between species. We kept ROHs with a Phred Score of at least 70 and with a minimum length of 100 Kbp. ROHs are considered long when above 1Mbp, medium between 500 Kbps and 1 Mbp and short below 100 Kbps. Visualization for all analyses was carried out in R v.4.3.2^66^ using ggplot2.61^71^.

### Migration

We estimated gene flow among different population groups within the Northern and Southern Catalonia lineages. Populations were grouped according to the genetic clusters retrieved in the PCA and, in some cases, further subdivided based on their geographic location. Because a minimum number of individuals per group is required to run the analysis, it was conducted only within Catalonian populations (localities 1–21). This approach also provides valuable insight into the status and potential origin of these populations. To do this, we generated a genepop file from Dataset 2 using the vcf2genepop function in the R package gwscaR^72^. Gene flow was then estimated with the function divMigrate in the R package diveRsity^73^. We calculated the number of effective migrants per generation (Nm), Jost’s D (D), and Nei’s GST (GST). The resulting matrix was plotted with the R package qgraph^74^, disregarding edges below 0.22.

### Demographic history

We inferred the demographic history of the species using Pairwise Sequential Markovian Coalescent (PSMC)^75^. This analysis was run on two individuals of *A. iberus* (one from the Northern and one from the Southern Catalonia lineages) and one *A. baeticus*, all three sequenced aiming at a 15X coverage. Heterozygous positions were obtained from bam files with Samtools v1.9^61^ and the data was filtered to assure a mapping and base quality > 30. A generation time (θ) of 0.5 and a mutation rate of 3.5 × 10^–9^ per bp per generation were assumed^76^. Ten bootstrap replicates were performed on each sample.

## Supplementary Information

Supplementary Information 1.

Supplementary Information 2.

Supplementary Information 3.

## Data Availability

The datasets generated and/or analyzed during the current study are available in NCBI/GenBank repository under BioProject PRJNA1320828, with accession numbers provided in Supplementary Table S1. [https://dataview.ncbi.nlm.nih.gov/object/PRJNA1320828?reviewer=mi56tduse3pe847n42t926s3e3].
