# Supplementary material for: Saving the locals: a conservation genomics approach to the endangered Spanish Toothcarp, Aphanius iberus (Valenciennes, 1846)
Source: Sci Rep. 2025 Dec 11;16:2150. doi: 10.1038/s41598-025-31909-y (PMC12808277; doi:10.1038/s41598-025-31909-y)
Supplement: Supplementary file 3 — Supplementary Information 3. [file 41598_2025_31909_MOESM3_ESM.pdf]

Supplementary results and discussion

The populations located in the Prat de Llobregat, in the Barcelona province (localities 7 and 8, situated within the airport facilities), have been controversial due to the nature of their historical background. Although the Spanish toothcarp was declared locally extinct in the area in the mid-20th century, a reintroduction program began in 1995 following the discovery and subsequent donation of a population by local aquarium hobbyists [1, 2]. Since 2008, the recovery project for the Spanish toothcarp in the Llobregat Delta has been underway, based on a captive breeding program developed in facilities located in a pine forest near the Barcelona airport, with progressive releases carried out in subsequent years in various ponds and coastal lagoons. In 2019, the Barcelona Zoo took the lead in coordinating this conservation initiative. This approach has proven successful, with populations now established in at least four sites comprising a system of isolated saline lagoons in the airport area. The results from this study indicate an admixed genetic profile between the lineages of Northern and Southern Catalonia, with the latter exhibiting a greater influence, likely due to its closer geographical proximity. In the absence of evidence to support or refute the hypothesis of a natural origin for this population and the preservation of the genetic line, the results demonstrated genetic congruence with the well-structured geographic gradient characteristic in the species [3, 4].

With regard to the populations situated between the Prat del Llobregat and the Ebro River Delta (localities 9-16), three genetically well-differentiated groups were identified. These are comprised by localities 9 and 11 (matching the Southern Catalonia lineage), localities 10, 13 and 14 (matching the Northern Catalonia lineage) and localities 12, 15, 16 and 17 (showing an admixed profile between both Catalonia lineages). The populations from localities 9, 10, 11, 13 and 14 have already been demonstrated to have an unnatural origin and, as a result, should be considered to have been translocated. In contrast, the admixed group is constituted by two geographically distinct populations. The northernmost of these mentioned populations (locality 12) is located in Salou and is named Tributaris-Sèquia Major. This population was not described until 1999 [2] and had never been genetically studied. The southernmost mentioned population is formed by three additional localities situated in close proximity to the Ebro Delta River: Sant Jordi d'Alfama, Torrent del Pi and Estany Tort (localities 15, 16 and 17, respectively). It has been established that an unidentified individual translocated the Spanish toothcarp into Torrent del Pi (locality 17) in 1987. Subsequently, the species was observed to undergo a natural colonization process, spreading to Sant Jordi d'Alfama and Estany Tort (localities 15 and 16, respectively), which are situated within an accessible distance for the species (approximately 1 km each from Torrent del Pi). The situation for locality 12, in Salou, is distinct as there is documented evidence that the species has been present there continuously since its first record in 1999 by García-Berthou and Moreno-Amich [2]. Moreover, the observed results are consistent with the geographic gradient that the species has demonstrated. The admixed profile of this population exhibited an intermediate profile between the Southern Catalonia lineage (as the Ebro Delta River) and the genetic profile shown by the captive-bred individuals from the Prat del Llobregat (localities 6-8), which provides compelling evidence of a well-structured geographic gradient (Fig. 2b and c). From a conservation perspective, the proximity of the introduced nuclei 15–17 to the native Ebro Delta populations (18–21) requires careful management, as they could either facilitate connectivity or pose a risk of introgression. We recommend targeted genetic monitoring and, if necessary, containment measures to safeguard the delta's

genetic integrity. The Ebro Delta populations themselves, demonstrated as native, represent ~98% of the species' distribution in Catalonia [5, 6] and are undergoing regression due to invasive species [7], making them a top conservation priority.

The population of Cabanes (locality 22) is located between the Levantine and the Southern Catalonia lineage. The admixed profile demonstrated by this population does not entirely align with the profile presented in previous studies. This discrepancy could be attributed to the absence of additional intermediate and passively natural populations, such as Peñiscola (between south Catalonia and Cabanes) and Grao de Castellón (between Cabanes population and Levantine lineage). Additionally, the inclusion of only one individual from each population may be a contributing factor. This is further corroborated by the fact that the populations of Murcia appear to be related despite their known differences [3, 4]. The analysis groups the population of Sax (locality 24) with Santa Pola (locality 25), San Pedro del Pinatar (locality 26), and Rambla de las Moreras (locality 27), which previous studies have differentiated and separated as distinct conservation units [3, 4]. Additionally, some of these populations, such as the one in Sax (locality 24), although of natural origin, have been maintained in captivity and may be subject to similar inbreeding effects as those observed in the populations from the Northern Catalonia lineage (Girona populations). Translocated populations pose a significant threat to those considered natural due to their genetic profile. The Adra population (locality 28) in Almería was recently identified as non-native and confirmed in this study [4]. Prior to the publication of this study, individuals from this population had already been translocated into some environments in nearby provinces, such as Granada and Málaga. This highlights the serious issue of undertaking reintroduction actions without a proper understanding of the true genetic origin of the source populations. Furthermore, these newly introduced populations are dangerously approaching the distribution range of the sister species *Aphanius baeticus*, which could potentially lead to more severe conservation challenges, including the risk of hybridization [8].

It can be surmised that the population from the Prat de Llobregat (localities 7 and 8), which have experienced growth in captivity at the Barcelona Zoo (locality 6), should be maintained and preserved, given the probable natural and unique genetic origin of the specimens, as demonstrated by the results. It is important to note, however, that the heterozygosity is relatively low and that the levels of ROHs are not optimal. A comparable scenario was observed at locality 12 (Tributaris - Sèquia Major population, in Salou). It's likely that this population has a natural origin and that its populations are well preserved, exhibiting similar heterozygosity levels. Nevertheless, in this case, less percentage of the genome is in ROHs. By contrast, the Ebro Delta populations (localities 18–21) show lower ROH levels and higher heterozygosity, indicating comparatively better genomic health. Given their presumed native origin and the fact that they represent the largest remaining continuous population of *A. iberus* in Catalonia, these populations hold exceptional conservation value and should be prioritized for protection. In contrast, translocated populations, such as localities 10, 13 and 14 and all other allochthonous populations (including those from the Barcelona province outside of the Prat de Llobregat lineage, localities 3–5), should not only be monitored but also considered for removal or containment measures where feasible, to prevent their dispersal and the genetic deterioration of native populations. These actions should be part of a coordinated management plan led by local authorities and institutions, aimed at safeguarding natural populations and avoiding the formation of unnatural, admixed profiles.

References

1. de Sostoa Fernández, F. J. *Biología de Aphanius iberus (Cuv. et Val., 1846) en el Delta del Ebro (NE ibérico)* [Doctoral dissertation, Universitat de Barcelona]. (1984)
2. García-Berthou, E., & Moreno-Amich, R. Ecología y conservación del fartet (*Lebias ibera*) en las marismas del Ampurdán (Cataluña). En M. Planelles-Gomis (Ed.), *Peces ciprinodóntidos Ibéricos: Fartet y Samaruc,* 151–161. Generalitat Valenciana, Conselleria de Medio Ambiente. (1999)
3. González, E. G., Cunha, C., Ghanavi, H. R., Oliva-Paterna, F. J., Torralva, M., & Doadrio, I. Phylogeography and population genetic analyses in the Iberian toothcarp (*Aphanius iberus* Valenciennes, 1846) at different time scales. *Journal of Heredity,* **109(3),** 253–263. https://doi.org/10.1093/jhered/esy003 (2018)
4. Nester, T. L., López-Solano, A., Perea, S., & Doadrio, I. Genomic population structure and diversity of the Endangered *Aphanius iberus*: Strategies for killifish conservation. *Conservation Genetics*, 1–15. https://doi.org/10.1007/s10592-024-01527-1 (2024)
5. López V., Franch, N., Pou, Q., Clavero, M., Gaya, N., & Queral, J.M. Atles dels peixos del delta de l'Ebre. Col·lecció tècnica, 3. Generalitat de Catalunya, Departament d'Agricultura, Ramaderia, Pesca i Medi Natural. Parc Natural del Delta de l'Ebre. 1a edició. 224p. (2012)
6. Clavero, M., Franch, N., Pou-Rovira, Q., & Queral, J.M. Disruption of salinity regimes in Mediterranean coastal wetlands and its impact on the coexistence of an endangered and an invasive fish. *Fishes Mediterr. Environ*, (2016)
7. Clavero, M., Franch, N., Bernardo-Madrid, R., López, V., Abelló, P., Queral, J.M., & Mancinelli, G. Severe, rapid and widespread impacts of an Atlantic blue crab invasion. *Marine Pollution Bulletin*, *176*, 113479. (2022)
8. Quilodrán, C. S., Montoya-Burgos, J. I., & Currat, M. Harmonizing hybridization dissonance in conservation. *Communications biology*, *3*(1), 391. https://doi.org/10.1038/s42003-020-1116-9 (2020)

Supplementary figures

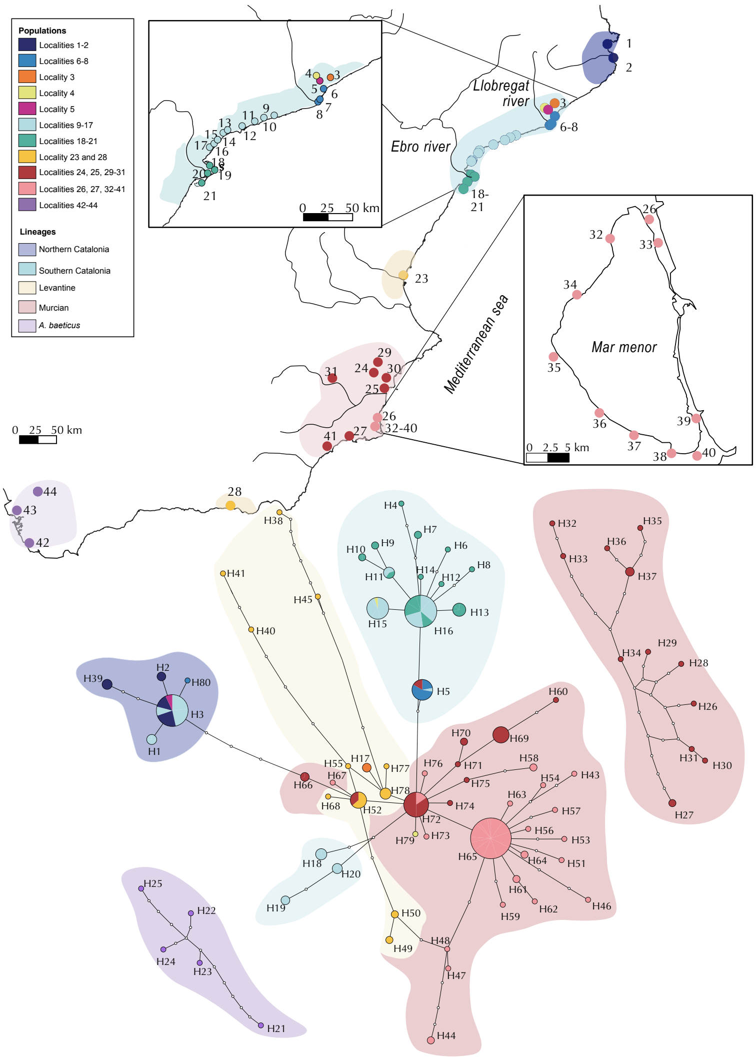

Supplementary Figure 1. Haplotype Network of the cytochrome b gene and map including the localities of all samples. Haplotypes are colored according to the locality as presented in the map and fully described on Table S2. Haplotypes are shaded to indicate the four genetic lineages: Northern Catalonia, Southern Catalonia, Levantine and Murcian.

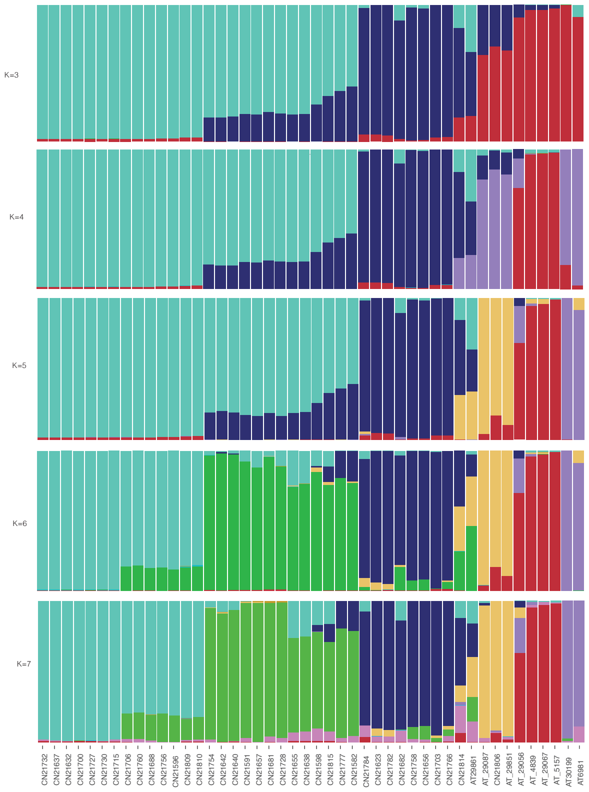

Supplementary Figure 2. Structf4 plots for K=3 to K=7 for all individuals, including outgroups.

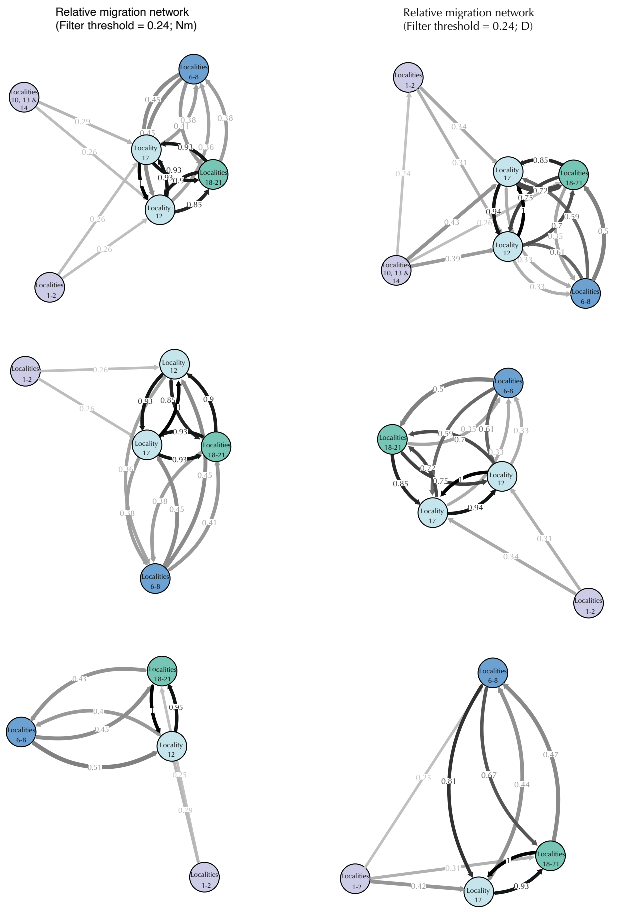

Supplementary Figure 3. Migration networks (number of migrants, Nm, and Jost's D) tested under different population sets, from all genetic groups to only confirmed native groups, to obtain clearer relationships without interference from translocated populations. Localities shown inside circles correspond to both lineages from Catalonia region: North Catalonia lineage: native: locs. 1–2 (Girona genetic group); translocated: locs. 10, 13, 14 (Girona genetic group). South Catalonia lineage: native: loc. 12 (Salou genetic group), locs. 6–8 (Prat de Llobregat genetic group), and locs. 18–21 (Ebro Delta River genetic group); translocated: loc. 17 (Salou genetic group).

Supplementary tables

Table S1. Table of specimens for which we obtianed whole genome sequencing data, their location, genetic lineage according to the PCA analysis, coverage and information on which individuals are included in each dataset. The coverage in parenthesis is to which the individuals of higher coverage were downsampled.

Table S2. Table of all specimens included in this study, their Haplotype for the Cytochrome b gene, sex, location and GenBank accession numbers. Samples in bold have been used for WGS.
